# A microfluidic platform for simultaneous quantification of oxygen-dependent viscosity and shear thinning in sickle cell blood

**DOI:** 10.1063/1.5118212

**Published:** 2019-11-15

**Authors:** José M. Valdez, Yvonne H. Datta, John M. Higgins, David K. Wood

**Affiliations:** 1 Department of Biomedical Engineering, University of Minnesota, Minneapolis, Minnesota 55455, USA; 2 Department of Medicine, University of Minnesota, Minneapolis, Minnesota 55455, USA; 3 Center for Systems Biology and Department of Pathology, Massachusetts General Hospital, Boston, Massachusetts 02114, USA and Department of Systems Biology, Harvard Medical School, Boston, Massachusetts 02115, USA

## Abstract

The pathology of sickle cell disease begins with the polymerization of intracellular hemoglobin under low oxygen tension, which leads to increased blood effective viscosity and vaso-occlusion. However, it has remained unclear how single-cell changes propagate up to the scale of bulk blood effective viscosity. Here, we use a custom microfluidic system to investigate how the increase in the stiffness of individual cells leads to an increase in the shear stress required for the same fluid strain in a suspension of softer cells. We characterize both the shear-rate dependence and the oxygen-tension dependence of the effective viscosity of sickle cell blood, and we assess the effect of the addition of increasing fractions of normal cells whose material properties are independent of oxygen tension, a scenario relevant to the treatment of sickle patients with blood transfusion. For untransfused sickle cell blood, we find an overall increase in effective viscosity at all oxygen tensions and shear rates along with an attenuation in the degree of shear-thinning achieved at the lowest oxygen tensions. We also find that in some cases, even a small fraction of transfused blood cells restores the shape of the shear-thinning relationship, though not the overall baseline effective viscosity. These results suggest that untransfused sickle cell blood will show the most extreme relative rheologic impairment in regions of high shear and that introducing even small fractions of normal blood cells may help retain some shear-thinning capability though without addressing a baseline relative increase in effective viscosity independent of shear.

## INTRODUCTION

Sickle cell disease (SCD) is a hematological disorder that affects millions worldwide with an expected 300 000 infants born with the disease per year.^1,2^ The major cause of morbidity and mortality in SCD is impaired blood flow culminating in vaso-occlusions, which includes contributions from hypoxia-induced changes in sickle blood cell mechanics, increased adhesion to endothelial cells, intravascular hemolysis, and increased inflammation.^3–5^ Although disease course often includes high rates of hospitalization and complications, there is a large range of clinical phenotypes including patient groups who experience completely benign disease. The mechanisms for this phenotypic diversity are largely unknown, and efforts to find useful genotypic or clinical predictors of benign prognosis have been unsuccessful.^6–8^ As such, developing tools and metrics to understand this clinical diversity is critical for both the management of patients and the development of new therapies.

Changes in red cell mechanics and whole blood rheology under hypoxic conditions are a hallmark of the disease, suggesting that the patient rheologic profile might provide a metric to explain the clinical phenotype.^9,10^ Most previous studies that have quantified sickle blood rheology have relied on cone-and-plate or Couette viscometers, which typically use surface driven flows, do not recapitulate the velocity profiles observed in the microvasculature, and can have artifacts in suspensions like blood.^11,12^ To address these challenges, we previously developed a microfluidic system to quantify sickle blood flow in a range of oxygen tensions and shear rates and in microchannels that mimic the size of vessels seen in the microvasculature.^13^ We demonstrated that oxygen-dependent changes in sickle blood flow are correlated with the clinical history, suggesting that such measurements might serve as biomarkers for disease severity. One limitation of these studies, however, was that they did not explore a range of shear rates and blood pressures. Such studies could reveal the changes in blood rheology, which are specific to various shear rate regimes in the microvasculature, under which regimes hypoxia most strongly impairs flow and contributes to vaso-occlusion. These measurements would also more fully quantify the patient rheologic phenotype and deepen our understanding of the clinical phenotype.

Thus, our objective in these studies was to quantify sickle blood effective viscosity over a range of physiologically relevant shear rates and oxygen tensions. One technical challenge to such studies, such as those using our previously published microfluidic platforms, is that impaired flow within the devices could lead to packing of red cells, thus complicating the interpretation of the effective viscosity. Here, we report the development of a new microfluidic platform that directly addresses this technical limitation to allow for absolute quantification of blood effective viscosity within physiologically sized microchannels and under controlled oxygen tension, blood pressure, and shear rates. We demonstrate the ability to profile sickle blood flow over a wide range of oxygen tensions and shear rates and to compare blood samples based on well-defined rheologic metrics such as the flow behavior index and viscosity. We also demonstrate the application of the platform to evaluate transfusion therapy and to provide *in vitro* comparison of therapeutic transfusion targets. With the creation of this model system, we provide a tool that allows us to probe some of the relevant physiological parameters that drive pathophysiology in SCD. In addition, we anticipate our model system can be used to fill the gaps of identifying markers that can objectively predict patient response to treatments, both those approved and those in development, and distinguish the characteristics that lead to observed patient diversity.

## RESULTS AND DISCUSSION

### Device oxygen transport modeling and validation

**FIG. 1.**
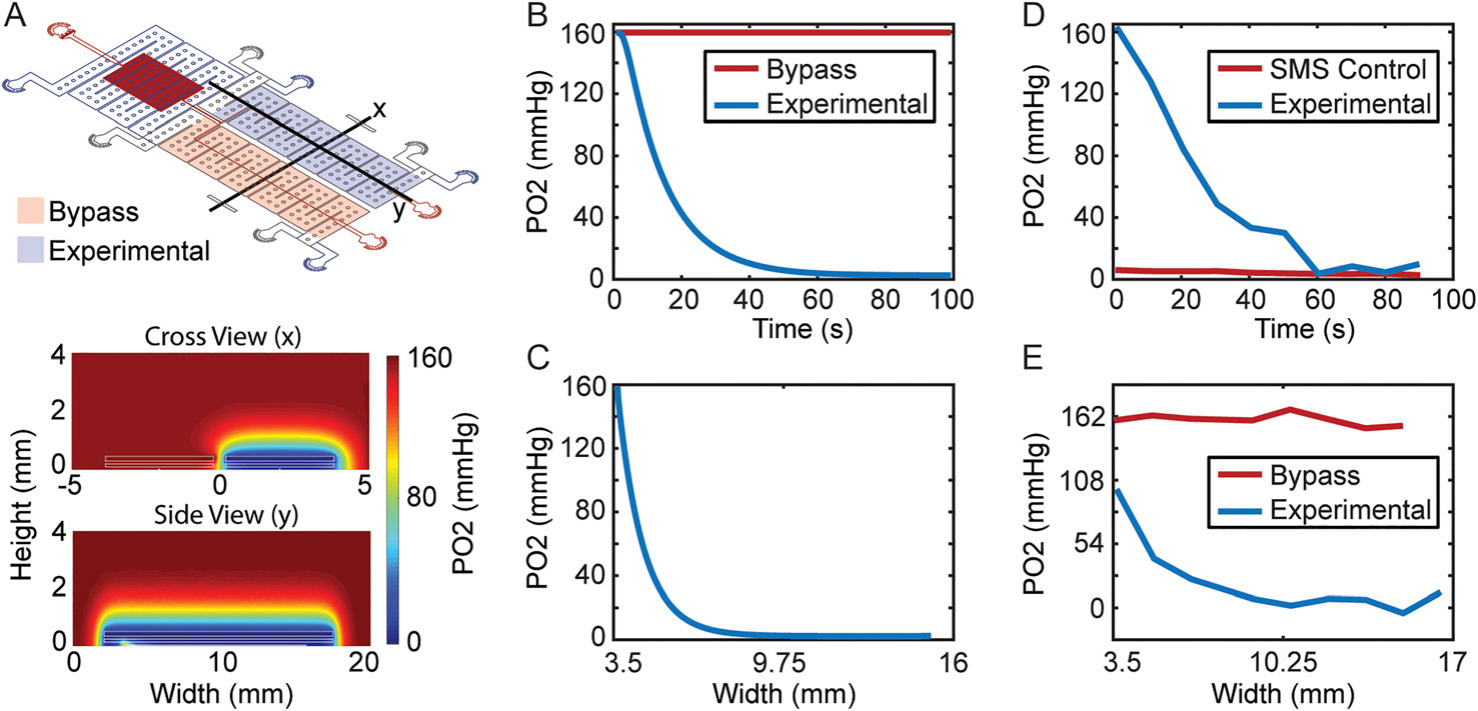
COMSOL oxygen transport modeling. (a) Cross-sectional and longitudinal device steady state views showing the device oxygen concentration profile. (b) Modeling probe at the crossview (x) location, showing that oxygen temporal response takes approximately 60 s to reach 0 mm Hg after switching off oxygen supply in the experimental section, while the bypass section remains unaltered at 160 mm Hg. (c) Spatial analysis shows device is at 0 mm Hg from approximately 7 mm to 15 mm length of the gas channel. (d) Experimental verification of oxygen temporal response using the oxygen sensitive dye Ru(bpy)3 shows the device reaches the 0 mm Hg Ru(bpy)3 SMS control after approximately 60 s but differs in the profile expected from the model. (e) Experimental verification of spatial oxygen response shows the device is at approximately 100 mm Hg at the location blood bends to enter the side view (y) section unlike the 160 mm Hg assumption in the model but still reaches 0 mm Hg for an approximately 8 mm length of the device experimental section. Oxygen tension was estimated using fluorescent calibrations at 0 mm Hg and 160 mm Hg using the oxygen-sensitive dye Ru(bpy)3.

A COMSOL model was created to simulate oxygen transport in the device design and experimentally confirmed using the oxygen sensitive luminescent dye Tris(4,7-diphenyl-1,10-phenanthroline)ruthenium(II) dichloride, abbreviated as Ru(bpy)3^22^ (Fig. 1). The device was modeled as a polydimethylsiloxane (PDMS) construct with a diffusion coefficient of 3.3 × 10^−5^ cm^2^/s and blood and hydration channels used the water diffusion coefficient of 4 × 10^−5^ cm^2^/s.^23^ Device external walls were modeled as air supply zones of 160 mm Hg oxygen tension due to being open to the environment except the bottom wall which was modeled as a no flux condition to represent the glass slide. Experimental and bypass gas reservoirs were modeled as gas supplies of nitrogen or air to verify each blood channel oxygen tension could be independently controlled, respectively. The model cross section and longitudinal section results showed that oxygen tension reached 0 mm Hg in the experimental zone, while bypass sustained 160 mm Hg and spatially the experimental blood channel was at 0 mm Hg for approximately 8 mm in length [Figs. 1(b) and 1(c)]. To experimentally verify oxygen concentrations in the device, a working solution of 1 mM Ru(bpy)3 phosphate-buffered saline (PBS) was perfused through the blood channel of the microfluidic device. As a 0 mm Hg oxygen control, 1 mM Ru(bpy)3 with a sodium metabisulfite (SMS) oxygen scavenger at saturating concentrations was used for calibration. The oxygen tension of the experimental gas reservoir was modulated corresponding to model properties, and respective luminescence intensity changes were detected using a Zeiss Axio Observer microscope. The results showed that experimental oxygen tension in the blood channel reached 0 mm Hg after about 60 s [Fig. 1(d)]. Spatial analysis of oxygen tension showed the bypass remained at ∼160 mm Hg oxygen tension fluorescence, while the experimental channel decreased to 0 from approximately 9.25 mm to 17 mm of the modeled length [Fig. 1(e)]. A notable difference is that the spatial oxygen tension started at ∼100 mm Hg vs 160 mm Hg for the experimental channel. This is due to our COMSOL probe not including the bend the channel makes to enter the gas coverage area and was plotted only along the y side view [Fig. 1(a)]. Despite this small discrepancy, the experimental zone has a similar 8 mm length under the desired oxygen tension, as simulated in the model.

### Bulk blood hemoglobin fractions alone do not determine the blood rheologic response to hypoxia

**FIG. 2.**
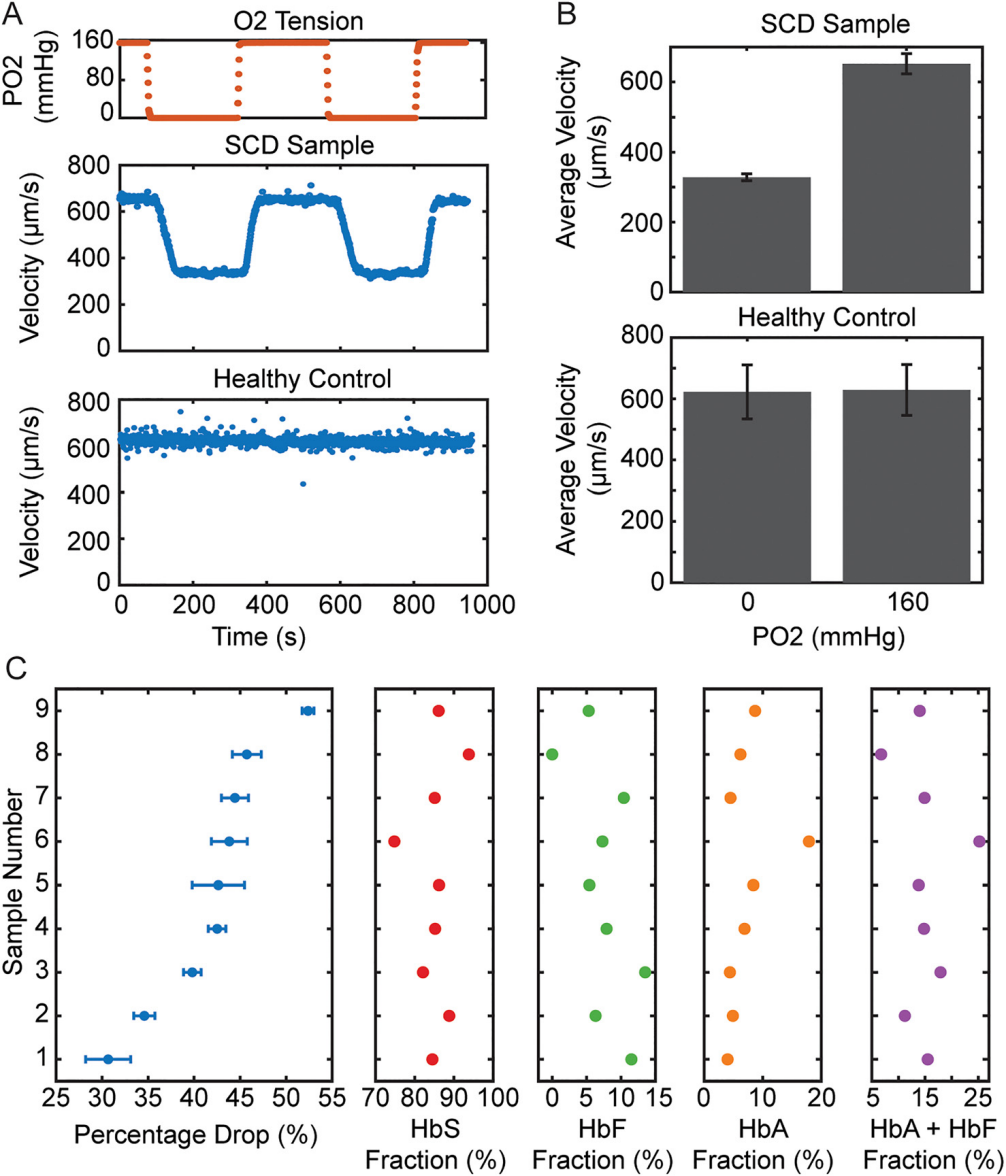
Velocity response of sickle cell blood to oxygen. (a) Oxygen tension was cyclically modulated between 160 and 0 mm Hg and blood velocity tracked and analyzed. A representative SCD sample followed by a healthy control are shown. (b) Representative average velocity at 160 and 0 mm Hg oxygen tension for sickle and normal blood samples. The error bars represent the measurement standard deviation for the multiple oxygen cycles measured and used to determine the average relative velocity drop. (c) These values were then compared using Pearson's coefficient to assess linearity with respect to hemoglobin fraction values obtained using high performance liquid chromatography, but no significant linear correlation was found relating hemoglobin fractions to rheology (p > 0.05 for all metrics using Pearson correlation), n = 9 patients.

The kinetics and extent of hemoglobin polymerization are determined by the HbS fraction in red blood cells. We measured blood flow steady state velocities in the experimental channel at 160 mm Hg and 0 mm Hg oxygen tensions for 9 samples from patients who had not recently been transfused (HbS fraction > 70%). We quantified the relative drop in velocity for each sample compared to the fractions of HbS and nonsickle hemoglobin present in each sample (Fig. 2). As we have previously observed,^13,17^ we saw that blood velocity for SCD patients strongly depends on oxygen tension unlike normal, healthy blood [Fig. 2(a)]. For each sample, the steady state velocity responses under oxygenated (160 mm Hg) and deoxygenated (0 mm Hg) conditions were evaluated and the fractional velocity change was quantified. The fractional velocity change among the samples was then compared with hemoglobin fraction data acquired for each sample using high-performance liquid chromatography (Tosoh G7, Tosoh Bioscience). We found no significant linearity relationships using the Pearson coefficient between the fractional velocity change and the fractions of hemoglobin A (0.38 HbA, p = 0.3012), hemoglobin F (−0.47 HbF, p = 0.2020), hemoglobin S (0.04 HbS, p = 0.9142), and the summation of hemoglobin A and F, the nonsickle variants (−0.04 HbF + HbA, p = 0.9225). Hemoglobin fractions thus were not considered strong drivers of observed blood rheology when the HbS fraction exceeded 70%. As the extent of hemoglobin polymerization is dependent on both the HbS concentration and the oxygen tension, small changes in the HbS fraction above 70% may not be associated with significant changes in flow characteristics in this range beyond 70%, consistent with some previous observations.^24^ In other words, at low oxygen tension, the effect of the HbS fraction on effective viscosity may saturate at levels of HbS lower than those found even in this patient cohort.

### Sickle cell blood remains shear-thinning under hypoxic conditions

**FIG. 3.**
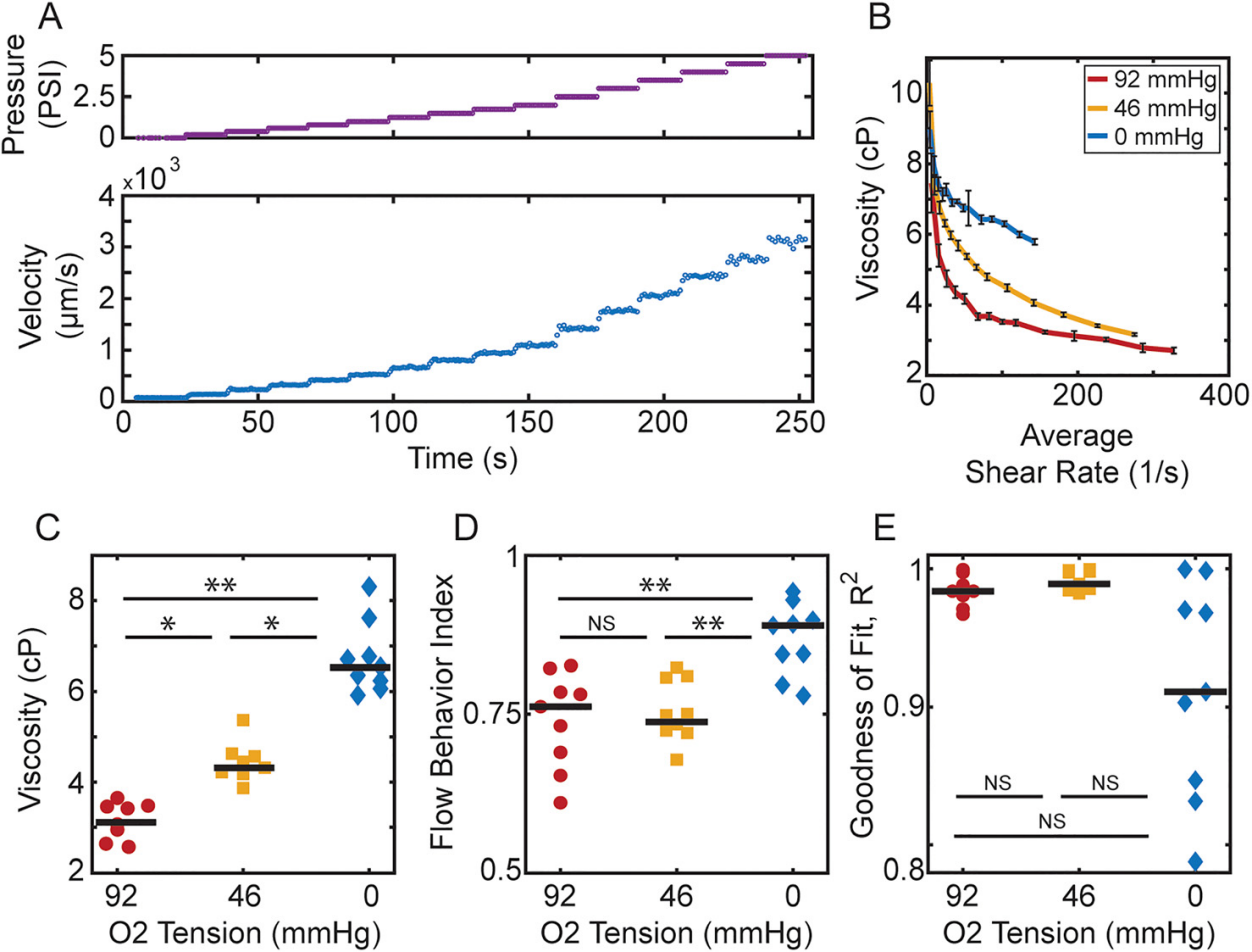
Blood viscosity measurement and quantification. (a) To acquire sample viscosity information at different oxygen levels, oxygen tension in the experimental zone is allowed to reach the steady state and then a series of pressure steps (top) are applied and velocity video data (bottom) collected. (b) Using our hydraulic circuit model (see Methods), viscosity vs average shear rate plots are generated for each oxygen tension analyzed. Error bars indicate the standard deviation of the measurement which on average was calculated using 18 video captures per pressure step. (c) For a set shear rate of 100/s, a significant increase in overall viscosity was found as the oxygen tension is decreased. (d) The flow behavior index is calculated by fitting the shear stress vs shear rate to a power law and extracting the exponent. Blood samples at 92 and 46 mm Hg oxygen tensions display similar flow behavior indices, despite the increased effective viscosity. At 0 mm Hg, flow behavior increases significantly, indicating reduced shear thinning. (e) This trend is further exemplified by decreased goodness of fit for the power law model at 0 mm Hg oxygen, indicating that blood no longer matches a conventional shear thinning model. All black bars in panels (c), (d), and (e) indicate the median in the distribution. Statistical significance was defined as: (^*^) p value < 0.05, (^**^) p value < 0.01, and (NS) no significance for p value > 0.05. The average shear rate is calculated as the maximum velocity at the channel midline divided by 7.5 *μ*m, half the channel width, n = 9 patients.

Because simple measurements of bulk effective viscosity do not reflect physiologically significant variation in blood characteristics, we used our microfluidic system to investigate the flow properties at finer levels of resolution. This microfluidic device enables us to measure a more complete rheological profile of sickle blood by modulating both the oxygen tension and the shear rate. This new device branches into “experimental” and “bypass” zones, which are maintained under controlled hypoxia and normoxia, respectively. In this setup, even when flow is impaired in the experimental zone under hypoxic conditions, normal flow is maintained in the bypass zone, mitigating cell packing and hematocrit (HCT) drift in the device. To quantify rheologic profiles, we varied driving pressure at each oxygen tension as well: blood oxygen tensions in the experimental zone were allowed to reach the steady state and then a series of pressure steps modifying the driving pressure were applied [Fig. 3(a)]. Based on channel dimensions, the average shear rates and channel impedances were calculated for each pressure and oxygen tension. As before, we first modeled the device using an equivalent hydraulic circuit (see Methods) to determine the effective viscosities at various shear rates and oxygen tensions [Fig. 3(b)].

Qualitatively, we observed that as expected, the viscosity of blood decreases with the increasing shear rate, indicating that the blood is shear thinning at each fixed oxygen tension. As shown in Fig. 3(c), we also observed that for a given shear rate, the blood effective viscosity significantly increases as we decrease oxygen tension (p < 0.05 between all oxygen tensions at 100/s). Interestingly, we observed that the shapes of the viscosity vs shear rate curves were similar at 92 mm Hg, [Fig. 3(b), red] and 46 mm Hg [typical capillary oxygen tension,^25^ Fig. 3(b), yellow], suggesting that there is a proportionally similar rate of decrease in effective viscosity as a function of increasing shear rate, even though the overall effective viscosity increases at 46 mm Hg. At 0 mm Hg [fully anoxic, Fig. 3(b), blue], the overall effective viscosity increases again, and there is also a qualitative change in the shape of the rheologic profile, suggesting a change in the shear thinning behavior of the blood.

### The degree of shear-thinning is greatly attenuated under extreme hypoxia

We quantified the overall degree of shear-thinning for a blood sample by calculating the slope of the log transformed relationship between shear stress and the shear rate. This slope, the flow behavior index, allows us to compare the degree of shear thinning across different blood samples and as a function of oxygen tension [Fig. 3(d)].^26^ Consistent with our qualitative observations, no significantly different flow behavior indices were found for 92 and 46 mm Hg oxygen tensions (p > 0.05). These results suggest that the first effect of lowering oxygen from ambient levels on an untreated sickle blood sample is to increase the absolute viscosity of the sample without affecting the degree of shear thinning in the blood. At 0 mm Hg, the flow behavior index increased significantly (p < 0.01 compared to 92 mm Hg or 46 mm Hg), corresponding to a reduction in shear thinning and higher overall impairment of blood flow [Fig. 3(d)].

Additionally, the goodness of fit (GOF) for the power law model had a larger spread at 0 mm Hg oxygen tension, compared to 92 and 46 mm Hg [Fig. 3(e)], consistent with a difference in the material properties at this oxygen tension and perhaps suggesting that a simple power law is no longer sufficient to model this fluid. The values however showed no significance (NS) when compared using a nonparametric Friedman test, and so additional analysis is required to determine the impact of the observed spread. Finally, we observed a spread among the unique patient samples measured at 92, 46, and 0 mm Hg oxygen tension, which is consistent with patient variability observed clinically: flow behavior indices of 0.76 ± 0.075, 0.74 ± 0.049, and 0.87 ± 0.056, respectively (mean ± SD).

### Rheologic profiles provide a means to evaluate transfusion therapy for individual patients

Blood transfusions are among the most common treatments for SCD patients and work at least in part by diluting SCD red cells with normal, healthy donor cells. A typical target value for transfusion is a 30% HbS fraction,^27^ comparable to the whole blood fraction for sickle cell trait (SCT), the heterozygous condition which is largely considered clinically benign,^28^ but a globally defined target for transfusion therapy may not be optimal for all patients.^29,30^ We used our platform to evaluate the shear-rate dependence and oxygen-tension dependence of blood from 2 patients at different simulated transfusion ratios. Blood was type-matched, and mixing ratios for each sample followed these standard values denoted as sickle hemoglobin percentage: 75%, 50%, 25%, 7.5%, and 0%.

**FIG. 4.**
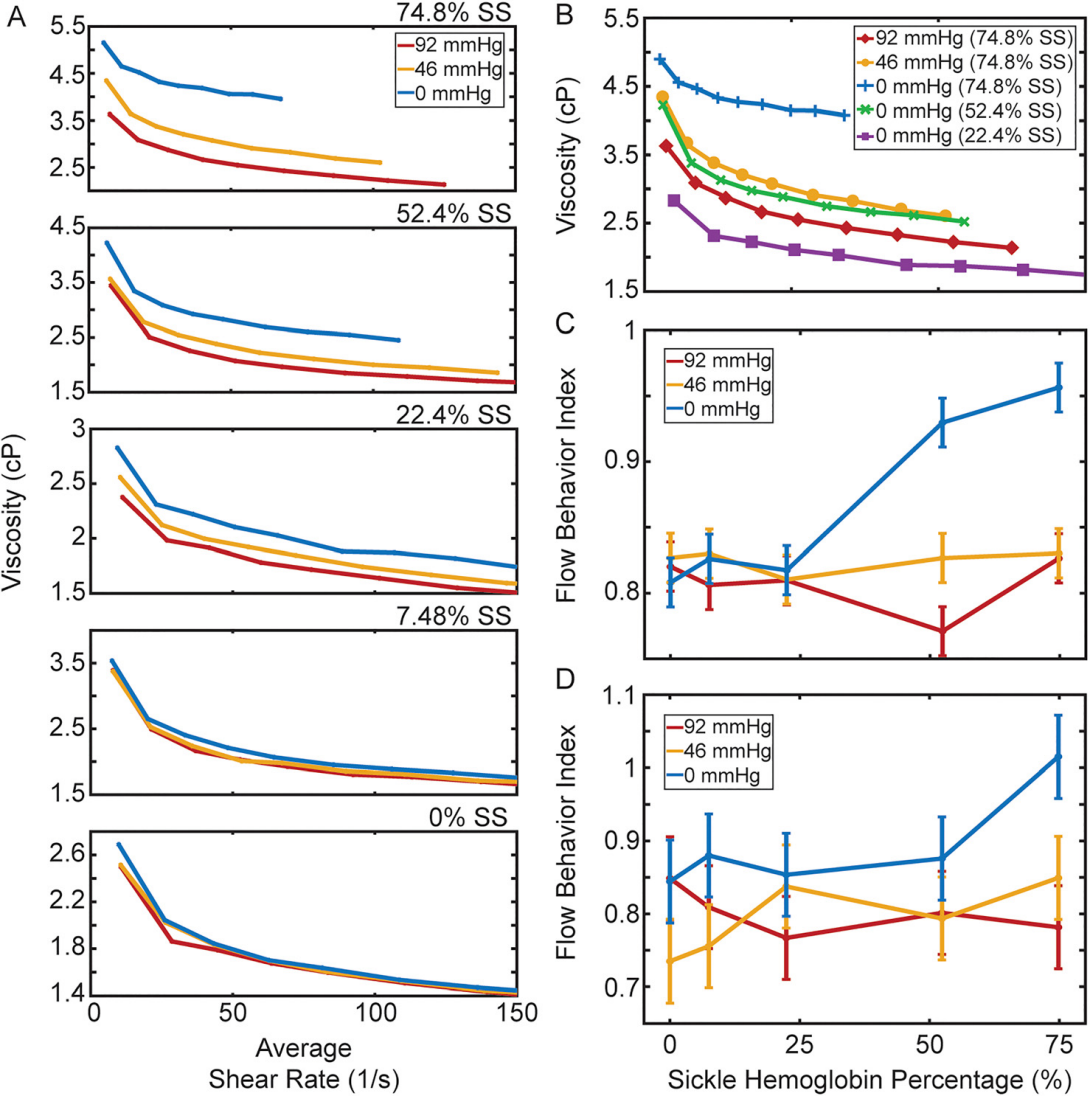
Simulated transfusion therapy experiment reveals increasing normal to sickle blood ratios leads to a loss of blood viscosity dependence on oxygen tension. (a) The viscosity vs average shear rate qualitatively shows a decreasing hemoglobin S concentration via transfusions leads to a loss of the viscosity dependence on oxygen levels indicated by curves collapsing onto each other. (b) Comparison of 0 mm Hg oxygen tension responses for different hemoglobin mix ratios reveals the viscosity benefits transfusions can offer. In this sample, decreasing the HbS percentage to 52.4% decreased the 0 mm Hg viscosity (green curve) below the 46 mm Hg viscosity at 74.8% HbS (yellow curve). Further decreasing HbS to 22.4% (purple curve) shows a blood response of 0 mm Hg viscosity even below the 92 mm Hg viscosity for a sample with 74.8% HbS (red curve). (c) Quantification of transfusion 1. At approximately 25% HbS concentration, the 0 mm Hg flow behavior index (blue) shows values comparable to 46 mm Hg and 92 mm Hg oxygen tensions, indicating a fluid behavior recovery response. (d) Unlike transfusion 1, a fluid behavior index drop is observed as early as 50% hemoglobin S concentration indicating patient differences. Generally, the metric can be optimized to be used as a tool to characterize patients specifically and provide individualized treatment guidance. Error bars represent the max spread between 92, 46, and 0 mm Hg at 0% HbS percentage as this sample should show no dependency on oxygen due to being a healthy control, and any variation would exist due to experimental error found in the device or noise in recording equipment, n = 2 patients.

As a representative dataset shows in Fig. 4(a), we observed that increasing ratios of normal blood to SCD blood led to decreased oxygen-dependent viscosity changes. Qualitatively, this relationship can be seen by the collapse of the viscosity curves onto each other as the normal blood ratio increases in the mixtures. It is important to note that a 0% HbS sample corresponds to a healthy HbA-only sample, but the viscosity is lower compared to the clinically reported values because we fix HCT at 25% to compare at the volume fraction typical for SCD patients including after transfusion. Increasing HCT to 45% will likely give similar values as those observed by others and clinically. Comparison of the 0 mm Hg oxygen tension viscosity response at different HbS percentages reveals the dramatic effect transfusions can have [Fig. 4(b)]. For this sample, decreasing HbS percentage to 52.4% at 0 mm Hg showed a viscosity behavior (green curve) comparable to 74.8% HbS at 46 mm Hg oxygen tension (yellow curve). We also computed flow behavior indices as a function of oxygen tension and HbS fraction [Figs. 4(c) and 4(d)]. On average, a 25/75 mixture leads to similar flow behavior indices at all oxygen tensions. However, there is a substantial difference between the absolute effective viscosities at each oxygen tension. While patient 1's blood sample [Fig. 4(c)] does not show a blood flow behavior index comparable to healthy blood until 25% HbS, the flow behavior index for patient 2's blood sample [Fig. 4(d)] resembles healthy blood at a HbS fraction as high as 50%. Thus, this measurement is able to reveal patient-specific differences in transfusions and could be used to generate patient-specific transfusion targets.

## CONCLUSIONS

In this work, we investigate the combined effects of varying shear rates and varying oxygen tensions on the flow behavior of sickle cell blood. We used a new microfluidic platform that allows oxygen concentration and shear rate modulation in physiological ranges. The device is able to control oxygen tension delivered to two different sections, preventing the packing of red blood cells, which would otherwise impact standard rheological measurements. We find evidence for two different changes in SCD blood flow. First, as oxygen tension falls, effective viscosity increases without affecting the relative shear thinning property of blood. At oxygen tensions below 46 mm Hg, both the viscosity profile and the flow behavior index show significant changes—the effective viscosity is increased, and the degree of shear-thinning is reduced. For transfusion therapy, we find increasing ratios of normal to sickle blood leads to improved rheological properties, and we find that these improvements are patient-specific, suggesting that these metrics could be helpful for clinical transfusion targets.

## METHODS

### Device design and fabrication

**FIG. 5.**
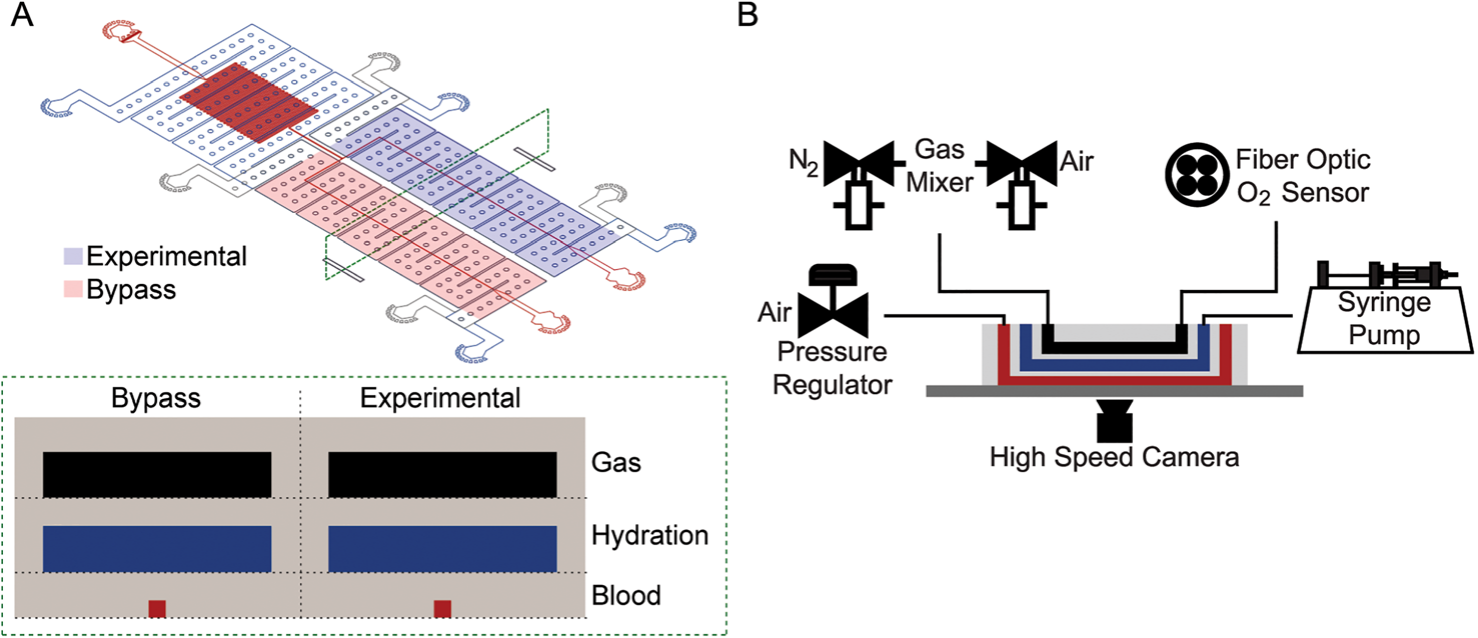
Device design and experimental setup. (a) The device is composed of three layers: a blood layer mimicking blood vessels (red), a hydration layer filled with phosphate-buffered saline to prevent blood dehydration (blue), and a gas layer used to control the partial pressure of oxygen in the oxygen layer (black). In addition, the device is subdivided into experimental and bypass zones. Controlled hypoxia in the experimental zone leads to the impairment of blood flow. The bypass zone, which is maintained at saturating oxygen partial pressures, mimics a cardiovascular branch and prevents red cell packing in the device when flow in the experimental zone is impaired. (b) A combination of control systems is utilized during experimental testing to perfuse blood (red), maintain blood hydrated (blue), mix and detect oxygen levels (black), and collect and analyze blood velocity data in real-time using custom MATLAB scripts.

We developed a microfluidic system specifically to measure changes in SCD blood flow while controlling the oxygen concentration and blood shear rate parameters, which are known to contribute to SCD pathologies.^14–16^ Inspired by our previous designs, the device is a multilayer polydimethylsiloxane (PDMS) structure with a blood, hydration, and gas layer construct each divided by a thin 100 *μ*m PDMS membrane (Fig. 5). For each of the three layers, standard photolithography using negative photoresist was used to create silicon master molds as previously reported.^17^ The blood and hydration silicon wafers underwent additional modifications to allow the creation of a thin 100 *μ*m PDMS membrane between each layer. For each wafer, four 100 *μ*m thick glass coverslips were attached to the wafer's outside perimeter equally spaced from each other. After silanization of the wafer, PDMS at a 10:1 elastomer/curing agent ratio was degassed and cast onto each layer. A transparency film was placed over the PDMS avoiding introduction of gas bubbles and a 10 lb metal weight placed on top of the wafer-PDMS-transparency film sandwich. The weight rests on the attached spacers compressing the PDMS to generate a 100 *μ*m PDMS membrane above the designed features. This setup was allowed to cure for 2 h on a hotplate set at 75 °C. The gas layer wafer was cast at a 4 mm PDMS height and cured in a standard convection oven for 2 h at 75 °C. To bond each layer, cured PDMS surfaces were plasma irradiated at an exposure time of 40 s, a power of 100 W, and an oxygen flow rate of 100 cc/min in a plasma cleaner. The final construct was then plasma bonded to a clean standard microscope glass slide to finalize device fabrication for use in experiments.

The blood layer has two zones: an experimental and bypass section both with an arteriole/venule sized microchannel with a cross-sectional area of 15 *μ*m × 15 *μ*m. The motivation for the branching channel design was to prevent blood packing during hypoxic conditions, which can lead to occluded channels. Under this scenario, the bypass zone is maintained at saturating oxygen partial pressures allowing blood to flow even when the experimental zone is occluded. The blood layer resistor was used as a pressure dissipater to achieve a range of velocity flow rates trackable by our computational algorithm. The hydration layer consists of two disconnected regions, one covering the experimental zone and another covering the bypass and resistor area, each 100 *μ*m tall. The purpose of the hydration layer was to hydrate the dry gas diffusing from each gas layer and prevent blood dehydration at an experimental temperature of 37 °C. Finally, the gas layer similarly consists of two disconnected regions both 150 *μ*m tall each covering the bypass and experimental zones, respectively. The two distinct gas zones allow independent control of gas delivery through each layer to control the oxygen tension for each blood channel.

### Blood handling and testing setup

Discarded whole blood from patients with sickle cell disease (SCD) and normal, healthy individuals was collected in EDTA vacutainers at the Massachusetts General Hospital and University of Minnesota Medical Center under Institutional Review Board approved protocols 2006P000066/PHS and STUDY0000003, respectively. All human subjects gave informed consent before participating in this study, or discarded specimens were used under an IRB-approved waiver of consent. Prior to testing samples, blood was stored at 4 °C for a few hours and up to three days. Previous studies have shown these storage conditions do not cause significant changes to rheological properties of blood.^18–20^ For all experiments, complete blood count tests provided hematocrit (HCT) and mean corpuscular hemoglobin concentration (MCHC) measurements. Using this information, sample plasma was removed and blood cells were resuspended using a phosphate-buffer saline solution to fix a 25% HCT. For simulated transfusion experiments, blood MCHC information was used to determine the relative transfusion necessary to achieve testing values of 0, 7.5, 25, 50, and 75% HbS. Transfusion experiment samples were similarly fixed at a 25% HCT and then mixed at appropriate ratios to allow for direct comparison between samples. Although 25% HCT is lower than physiologic for genotype HbAA adults, it reflects SCD patients' typical HCT, and it is also relevant for the transfused samples, as one clinical goal in transfusion therapy is to avoid the increase in HCT much beyond 25%. To measure blood rheological properties, the microfluidic device was mounted on a temperature regulated Zeiss Axio Vert microscope set at 37 °C and resuspended blood perfused using an electronic pressure regulator (PCD-15PSIG, Alicat Scientific). Phosphate-buffered saline was perfused through the hydration channel using a syringe pump (NE-500, New Era Pump Systems) to prevent dehydration of the blood. Oxygen gas tension was controlled by mixing 160 mm Hg oxygen gas (21% O_2_, 5% CO_2_, balance N_2_) with 0 mm Hg oxygen gas (5% CO_2_, balance N_2_) using a previously developed solenoid valve gas mixing setup that periodically duty cycles the two gases.^17^ Through the duration of the experiment, oxygen gas tension was continuously monitored on a device using a fiber optic oxygen sensor (NeoFox-GT, Ocean Optics). Video data of blood cells flowing in the experimental device channel were collected using a high-speed camera (GS3-U3‐23S6M-C, FLIR) and analyzed using a custom MATLAB point tracker algorithm. The MATLAB point tracker is based on the Kanade-Lucas-Tomasi (KLT) algorithm that lays points on images based on spatial intensity differences and tracks them through a set of frames. Using this framework and known device dimensions, frame by frame changes can be measured to calculate the velocity of blood in our channels.

### Microfluidic hydraulic circuit analysis

Based on Eq. (1) for laminar flow, we can write the total pressure drop in the device ($\Delta p_{\mathrm{total}}$) is equal to the product of the total flow rate ($Q_{\mathrm{total}}$) and the equivalent hydraulic resistance of the device ($R_{\mathrm{hyd}}$).^21^ To verify laminar flow in our system, we calculated the Reynolds number (Re) using Eq. (2), where ρ is the blood density, $\left\langle u\right\rangle$ is the blood mean velocity, L is the hydraulic diameter of the channel, and μ is the blood dynamic viscosity. Using blood metrics of $\rho =1060\;\frac{\mathrm{kg}}{m^{3}}$ and $\mu =0.004\;\frac{\mathrm{kg}}{m\;s}$ plus device and experimental characteristics of L=15 μm and ${\left\langle u\right\rangle }_{\text{max}}=3500\;\frac{\mu m}{s}$, which represents the maximum velocity observed in our experiments, we calculate a Reynolds number Re = 0.014, well below the transition to turbulent flow,

$$\;\Delta p_{\mathrm{total}}=Q_{\mathrm{total}}R_{\mathrm{hyd}},$$ (1)

$$\;Re=\frac{\rho \left\langle u\right\rangle L}{\mu },$$ (2)

$$\;R_{\mathrm{hyd}}={R_{1}+R_{2}+R_{3}=c}_{1}\eta_{1}+\frac{1}{\frac{1}{c_{2}\eta_{2}}+\frac{1}{c_{3}\eta_{3}}}.$$ (3)

**FIG. 6.**
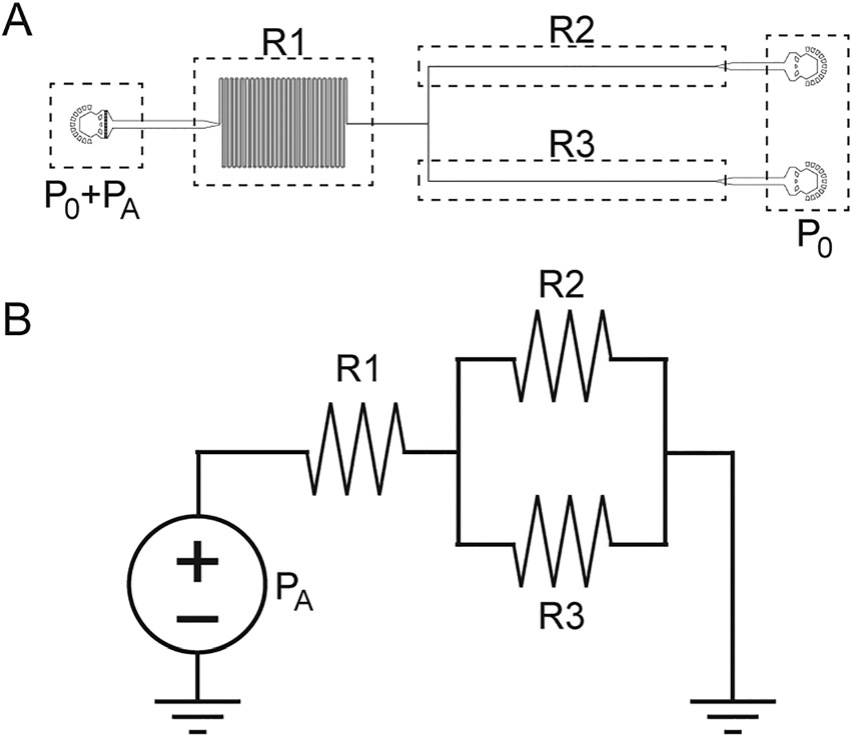
Blood section of our device with (a) labeled resistances and pressures and (b) its analogous view as an electrical circuit assuming Re < 1.

The hydraulic resistance [Eq. (3)] is determined from an equivalent circuit representation of our microfluidic system (Fig. 6). Because the total pressure drop and flow rates are directly measured, the total resistance can be calculated. Equation (3) shows that the resistance of the device ($R_{\mathrm{hyd}}$) is equal to the sum of the resistances in the device obtained using parallel and series resistor rules. Each resistance is simplified to a constant ($c_{1},\;\;c_{2},\;c_{3}$) that depends on the known resistor geometry multiplied by the effective viscosity at each section ($\eta_{1},\eta_{2},\eta_{3}$). The viscosities are simplified to piecewise values at each section due to resistors being much longer compared to transition regions at such low Reynolds numbers. The constants are calculated using the standard resistance formulas for square and rectangular channels expressed in Eqs. (4) and (5), respectively, leaving only the effective viscosities unknown. The variables in Eqs. (4) and (5) are defined as follows: h is the channel height, L is the hydraulic diameter of the channel, and w is the channel width.

$$R=\frac{12\eta L}{0.422h^{4}},$$ (4)

$$\;R=\frac{12\eta L}{1-\frac{0.63h}{w}}\times \frac{1}{h^{3}w}.$$ (5)

Combining Eqs. (1) and (3), we are left with one equation and three unknown effective viscosities shown in the following equation:

$$\;p_{\mathrm{total}}=Q_{\mathrm{total}}\left(c_{1}\eta_{1}+\frac{1}{\frac{1}{c_{2}\eta_{2}}+\frac{1}{c_{3}\eta_{3}}}\right).$$ (6)

To simplify the problem, we linearize the model by assuming that the relative velocities in each of the 3 resistors will be proportional to the cross-sectional area. We justify this assumption because of the low Reynolds number, the steady state conditions within the device, and the relatively small differences in the shear rate between different regions of the device. With this simplification, we know the ratio of the shear rates between the different sections of the device. Moreover, our measurements of device impedance (velocities at a given overall pressure, Fig. 3) show the ratio of the effective viscosities in the various parts of the device based on the relative shear rates—i.e., $\eta_{1}=A\eta_{3}$ and $\eta_{2}=B\eta_{3}$. Thus, all three effective viscosities can be written in terms of a single effective viscosity and two constants that are functions of the measured shear rate. Finally, using Eq. (7), we can compute the effective viscosity in the experimental zone, using known geometrical and measured parameters with which the experimental data were collected. This is done by solving Eq. (7) generating relationship factors A and B between each shear rate step to solve for the overall viscosity curve,

$$\;\eta_{3}=\frac{\frac{P_{\mathrm{total}}}{Q_{\mathrm{total}}}\left(\frac{1}{c_{2}B}+\frac{1}{c_{3}}\right)}{1+\frac{c_{1}A}{c_{2}B}+\frac{c_{1}A}{c_{3}}}.$$ (7)

### Statistical analysis

Linearity between the fractional velocity change and hemoglobin fractions was evaluated using the Pearson correlation coefficient, which indicates total positive linear correlation to total negative linear correlation from 1 to −1. To compare rheological properties across different measurements on a set of patient samples, we used a nonparametric Friedman's test with multiple comparison testing using a Bonferroni correction. Significance was set at p = 0.05 for all statistical tests performed.
